# Factors influencing engagement in online dual practice by public hospital doctors in three large cities: A mixed-methods study in China

**DOI:** 10.7189/jogh.13.04103

**Published:** 2023-09-22

**Authors:** Duo Xu, Yushu Huang, Sian Tsuei, Hongqiao Fu, Winnie Yip

**Affiliations:** 1 Institute of Population and Labor Economics, Chinese Academy of Social Sciences, Beijing, China; 2 Department of Health Policy and Management, School of Public Health, Peking University Health Science Center, Beijing, China; 3 Department of Global Health and Population, Harvard T.H. Chan School of Public Health, Boston, Massachusetts, USA; 4 Department of Family Practice, University of British Columbia, Vancouver, British Columbia, Canada; 5 Center for Health Policy and Technology Evaluation, National Institute of Health Data Science at Peking University, Beijing, China

## Abstract

**Background:**

In the digital age, a rising number of public sector doctors are providing private telemedicine and telehealth services on online health care platforms. This novel practice pattern – termed online dual practice – may profoundly impact health system performance in both developed and developing countries. This study aims to understand the factors influencing doctors' engagement in online dual practice.

**Methods:**

Using a mixed-methods design, this study concurrently collects quantitative demographic and practice data (n = 71 944) and semi-structured interview data (n = 32) on secondary and tertiary public hospital doctors in three large Chinese cities: Beijing, Shanghai and Guangzhou. We use the quantitative data to examine the prevalence of the online dual practice and its associated factors via the binary logit regression model. The qualitative data are used to further explore associated factors of online dual practice via thematic analysis. The findings about associated factors from the two parts were merged using the categories of personal, professional, and organisational characteristics.

**Results:**

Our quantitative analysis shows that at least 47.1% of public hospital doctors are involved in online dual practice. The shares in Beijing, Shanghai, and Guangzhou are 43.7%, 53.1%, and 44.8%, respectively. This practice is more prevalent among doctors who are male, senior, and non-managerial. Different specialties, hospital ownership, hospital levels, and locations are also significantly associated with this practice. The qualitative analysis further suggests that financial returns, perceived effectiveness of telemedicine, and hospital directors’ attitude towards telemedicine may affect doctors’ engagement with online dual practice.

**Conclusions:**

Online dual practice is prevalent among doctors at tertiary and secondary public hospitals in Beijing, Shanghai, and Guangzhou. Personal, professional, and organisational characteristics are all associated with doctors’ choice to engage in online dual practice. The findings in this study provide implications for promoting telemedicine adoption and developing relevant regulatory policies in China and other countries.

The shortage of health professionals is a significant barrier to universal health coverage for developing countries [1]. The movement of skilled health professionals to private institutions could exacerbate the shortage in the public sector [2,3]. Since the early 2000s, private Internet companies have established an increasing number of online health care platforms worldwide, enabling patients to access distant doctors via texts, audio, or videoconferences. The coronavirus disease 2019 (COVID-19) pandemic further boosted their developments [4,5]. Although these private platforms have the potential to revolutionise health care delivery [6-8], they might compete with public institutions for health professionals. In some countries where the public sector plays an important role in health care service delivery, doctors from public health care facilities are increasingly providing direct-to-consumer services on private telemedicine platforms – a phenomenon called “online dual practice” [9].

Although online dual practice is rising in popularity – emerging at least in China, France, India, Malaysia, and the Philippines (Table S1 in the **Online Supplementary Document**) – it is currently poorly understood. While the literature has well studied the prevalence, influential factors, systematic impacts, and regulatory options about dual practice in brick-and-mortar health care facilities [3,10-13], much less is known about the new form of dual practice on the Internet. Investigating what drives doctors’ pursuit of such practice patterns is worthwhile because concerns are emerging that such practice patterns introduce new physician incentives, which can generate unintended, potentially adverse, impacts on the health system [14]. For example, public hospital doctors may be distracted from their designated work due to online service provision, and therefore health system performance is likely to be undermined [9,15]. Given the global shift towards online health care services, understanding factors influencing engagement in online dual practice would offer policy implications for designing appropriate regulatory policies to mitigate adverse consequences of such practice.

As a country where public hospitals play a dominant role in the delivery system [16], China offers a particularly unique setting to undertake such a study. In the past decade, the telemedicine and telehealth industry in China has taken off with favourable policies from the central government and substantial investments from private internet companies [17,18]. A dozen private telehealth platforms have since emerged, and they constitute a large market share in online service provision [19], as shown in Table S2 in the **Online Supplementary Document**. These private platforms primarily draw on doctors from Chinese public hospitals. These doctors typically work as independent contractors with the freedom to set their own prices and share their earnings from online service provision with the platforms, thus forming the online dual practice phenomenon. A previous study showed that more than 16.5% of public hospital doctors in China engaged in online dual practice by the end of 2020 and such practice covered more than half of senior public hospital doctors in some large cities [9].

This study aims to capture the factors that lead public hospital doctors to engage in online dual practice, utilising data from three major Chinese cities. This research will examine personal, professional, and organisational factors that may influence doctors' decisions regarding online service provision, using quantitative and qualitative methods. The analysis will provide insight into the drivers of telemedicine adoption and corresponding regulatory policies for China and other developing countries.

## METHODS

### Mixed-methods design

**Figure 1:**
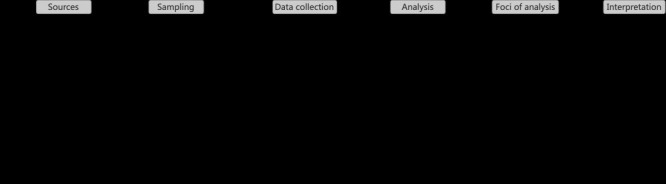
An illustration of the mixed-methods design used in this study. We employed a concurrent mixed-methods design to leverage the strengths of both quantitative and qualitative branches. The quantitative part sampled key characteristics of public hospital doctors at secondary and tertiary public hospitals in three major Chinese cities (Beijing, Shanghai, and Guangzhou). Characteristics such as gender, age, education, seniority, and specialty were collected by Haodf.com. This part also exhaustively sampled online doctors from four leading third-party online health care platforms (Haodf.com, WeDoctor, Chunyu Doctor and Ping’an Health) to identify the doctors engaged in online dual practice.

In this study, online dual practice is defined as public hospital doctors rendering online medical services on third-party private online platforms [9]. We employed a mixed-methods design to examine factors associated with the engagement of online dual practice to develop a richer understanding of this phenomenon. The mixed-methods approach enriches the understanding of motivational consideration among public hospital doctors, and the dual methodology approach adds rigor to the overall interpretation by informing and supporting each other [20-23]. We used the concurrent approach to optimise the efficiency of data collection on public hospital doctors in three large Chinese cities: Beijing, Shanghai, and Guangzhou (**Figure 1**). We purposefully selected these cities to investigate public hospitals’ adoption of telemedicine because they were three of the most important medical hubs as well as the most populous and technologically advanced cities in China. Online dual practice was more common in those cities than in other areas, as shown in a previous study [9]. Investigation on those doctors may thus more strongly reveal their motivations for engaging online dual practice. In addition, we focused on public hospital doctors at secondary and tertiary public hospitals in these cities since they delivered almost 50% of online health care services on private platforms [24] and their information was completer and more available.

The quantitative and qualitative data were obtained separately from the same cohort of doctors. The results were integrated at the interpretation stage. In the quantitative part, we first obtained a data set from Haodf.com, which contained extensive information on all public hospital doctors at secondary and tertiary public hospitals in these three cities. We then used web crawlers on four leading private online health care platforms (i.e. Haodf.com, WeDoctor, Chunyu Doctor, and Ping’an Health) to identify online dual practitioners. These two data sets were used to jointly examine the prevalence of the online dual practice and its influencing factors. In the qualitative part, we conducted in-depth semi-structured interviews with 32 public hospital doctors from three cities to further identify and explore associated factors of engagement in online dual practice. Results from the two parts enriched the understanding of what drove providers to pursue online dual practice. They also triangulated each other and improved the overall validity of the findings. We merged findings on associated factors from the two parts using the categories of personal, professional, and organisational characteristics that were identified in previous studies [3,25].

### Quantitative component

#### Data collection

We obtained a cross-sectional data set from Haodf.com on public hospital doctors at secondary and tertiary public hospitals in Beijing, Shanghai, and Guangzhou in December 2020. Since its inception in 2006, Haodf.com has been devoted to providing the public with accurate information on public hospital doctors who provide in-person services, especially public hospital doctors in medical hubs. As a result, Haodf.com created a data set of Chinese public hospital doctors that contained extensive information including doctors’ gender, age, seniority, education, specialty, and affiliated hospitals. This data set was regularly updated by representatives from Haodf.com through information from bulletin boards at public hospitals and interviewing administrative staff. It should be noted that this data set covered all public hospital doctors who provided in-person outpatient services, regardless of whether they delivered online health care services on Haodf.com (Figure S1 in the **Online Supplementary Document**). Doctors who had medical licenses but were unavailable to see patients were omitted. The data set used in this study included 28 834 public hospital doctors in Beijing, 23 192 in Shanghai, and 19 918 in Guangzhou.

At the same time, we obtained information on public hospital doctors who were engaged in online dual practice from the websites of four online health care platforms. We used a Python-based web-crawler to systematically scrape and extract data from the websites at the end of 2020. Only online dual practitioners in the three cities were included in the data set. We defined online dual practitioners as those who provided at least one type of the following health care services via direct-to-consumer telemedicine: text-and-image consultations, phone-call consultations, video consultations, and online appointments for in-person visits. The web-crawled data set covered a total of 33 863 online dual practitioners at secondary and tertiary public hospitals in Beijing, Shanghai, and Guangzhou; among them, 20 634 doctors were exclusively from Haodf.com, 6255 were exclusively from WeDoctor, 295 were exclusively from Chunyu Doctor, 1461 were exclusively from Ping’an Health, and 5218 doctors worked on at least two platforms.

### Statistical analysis

Taking these two data sets, we first estimated the prevalence of online dual practice among public hospital doctors at secondary and tertiary public hospitals in three large cities. Estimates of prevalence by doctors’ seniority and cities were also calculated based on doctors’ professional and location information captured in the data. The 95% confidence intervals (CIs) on the prevalence measures were calculated using the Wald method. To avoid double-counting, doctors working on multiple platforms were counted only once. As we have 71 944 observations, the sample size passed the testing with a confidence level of 95% and a small margin of error (±1%) [26].

We then quantitatively tested the association between online dual practice and doctors’ characteristics. We first merged the two data sets based on the doctors’ names, seniority, specialties, and affiliated public hospitals. As the dependent variable, online dual practice, was a binary variable, we then used the logit regression model to explore factors influencing doctors’ inclination to engage in online dual practice. The dependent variable was coded as one if a public hospital doctor provided at least one type of online health care services and zero otherwise. We focused on the doctors’ personal and professional characteristics, as well as their organisational settings in the process of data collection. Personal and professional characteristics included age, gender, education, seniority, clinical specialty, and administrative position. Variables measuring organisational settings included hospital level, hospital location, and ownership. The status of military hospitals was paid attention to because they served a substantial share of patients in these cities, and they might present different patterns of online dual practice. We reported odds ratios (ORs) and the average marginal effect (AME) for each variable. Stata 16.0 was used in the quantitative analyses on the prevalence and influential factors.

### Qualitative component

Between November 2020 and April 2021, we conducted in-depth semi-structured interviews with public hospital doctors in these three large cities. We conducted a heterogeneous purposive sampling that maximised respondents’ variations in gender, age, specialty, hospitals, and geographical locations, because these characteristics may affect doctors’ practice preferences. We generated a shortlist of potential respondents and then contacted them by sending messages to ask for their consent to participate. We recruited new participants until information saturation was achieved [27]. In the end, 32 public hospital doctors participated in the interview. The characteristics of the interviewees are shown in **Table 1**. Among them, 22 participants engaged in online dual practice. Twenty-one participants were male, 18 were from tertiary hospitals, and 13 were chief physicians. Eleven interviewees were from Beijing, 12 were from Shanghai, and nine were from Guangzhou. 12.5% of interviewees were hospital directors or vice hospital directors, and 21.9% of interviewees were department directors.

**Table 1:** Characteristics of interview participants

Characteristics	N	%
**Status of online DP**		
Engaged	22	68.8
Not engaged	10	31.3
**Gender**		
Male	21	65.6
Female	11	34.4
**Region**		
Beijing	11	34.4
Shanghai	12	37.5
Guangzhou	9	28.1
**Hospital Level**		
Tertiary	18	56.3
Secondary	14	43.8
**Seniority**		
Chief physician	13	40.6
Associate chief physician	10	31.3
Attending physician	7	21.9
Resident physician	2	6.3
**Specialty**		
Internal medicine	9	28.1
Surgery	7	21.9
OB/GYN	6	18.8
Paediatrics	4	12.5
Dermatology	3	9.4
Others*	3	9.4
**Position**		
Hospital director	4	12.5
Department director	7	21.9
No managerial positions	21	65.6

DP – dual practice, OB/GYN – obstetrics and gynaecology

*Other departments include departments like rehabilitation medicine, stomatology department, Traditional Chinese Medicine, and radiology department.

We drafted an interview guide and asked respondents about personal, professional and organisational factors that influenced doctors’ decisions to engage in online dual practice. The guide was originally in Chinese and an English translation was provided in Table S3 in the **Online Supplementary Document**. The interviews were carried out by the first and second authors, both of whom had training in qualitative methods and health policy research. We piloted the interview guide when interviewing the first three doctors and made minor revisions in the following interviews. All interviews were conducted in Mandarin over the phone and typically lasted from 15 to 40 minutes.

All interviews were transcribed verbatim in Chinese. We used a thematic approach with the assistance of the NVivo 12 software to analyse the data. We began our analysis by deductively developing initial codes from the literature [3,28,29]. The first author and the corresponding author independently coded the first eight transcripts using the initial codes. Then new codes were developed inductively to capture emerging concepts. Divergences were discussed until a consensus was reached. The senior author provided tip-breaking opinions during this process. The refined codes were applied to the remaining transcripts and were used to distil key themes. We paid close attention to opposing opinions among respondents. Once the analysis was complete, we translated illustrative quotes into English for the manuscript.

### Patient and public involvement

No patients or members of the public were directly involved in the design, data collection or analysis of this research.

## RESULTS

### Descriptive statistics

Our sample contained 71 944 doctors at secondary and tertiary public hospitals in Beijing (40%), Shanghai (32%), and Guangzhou (28%), as shown in **Table 2**. Eighty-five percent of them were from tertiary hospitals, and 7% were from military hospitals. Sixty-six percent were male and the average age was 41.6 years old. Among these doctors, 35% had a doctoral degree, and 52% were senior doctors (chief or associate chief physicians). Approximately 6% of these public hospital doctors were the directors of their department and 1% of them were hospital directors or vice hospital directors. **Table 2** also shows that there are substantial differences between dual practitioners and non-dual practitioners. Online dual practitioners accounted for 47% of the doctors. They were generally older, more senior, more educated, more likely to be from tertiary hospitals, and less likely to be from military hospitals.

**Table 2:** Descriptive statistics of the quantitative part

	Total* (n = 71 944)		Online DP† (n = 33 863)		Non-online DP (n = 38 081)	
	**Mean**	**SD**	**Mean**	**SD**	**Mean**	**SD**
**Individual characteristics**						
Gender						
*Male*	0.66	0.47	0.69	0.46	0.63	0.48
*Female*	0.34	0.47	0.31	0.46	0.37	0.48
Education	41.59	6.90	43.49	6.96	39.90	6.37
*College*	0.14	0.35	0.10	0.30	0.17	0.38
*Master degree*	0.51	0.50	0.43	0.50	0.59	0.49
*Doctoral degree*	0.35	0.48	0.47	0.50	0.24	0.43
**Professional characteristics**						
Seniority						
*Chief*	0.19	0.39	0.29	0.45	0.11	0.31
*Associate chief*	0.33	0.47	0.35	0.48	0.31	0.46
*Attending*	0.34	0.47	0.27	0.45	0.40	0.49
*Resident*	0.14	0.35	0.09	0.29	0.19	0.39
Position						
*Department director*	0.06	0.24	0.06	0.23	0.06	0.24
*Hospital manager*	0.01	0.09	0.01	0.07	0.01	0.10
Department						
*Internal medicine*	0.20	0.40	0.20	0.40	0.19	0.39
*Surgery*	0.22	0.41	0.27	0.44	0.18	0.38
*OB/GYN*	0.06	0.24	0.06	0.24	0.06	0.24
*Paediatrics*	0.06	0.24	0.06	0.24	0.06	0.24
*Dermatology*	0.02	0.14	0.02	0.15	0.02	0.13
*Psychiatric*	0.02	0.13	0.02	0.13	0.02	0.13
*TCM*	0.13	0.33	0.13	0.34	0.13	0.33
*Others*‡	0.30	0.46	0.24	0.43	0.34	0.48
**Organisational characteristics**						
Ownership						
*Government & SOE*	0.93	0.26	0.98	0.13	0.88	0.32
*Military*	0.07	0.26	0.02	0.13	0.12	0.32
Hospital level						
*Secondary*	0.15	0.36	0.12	0.32	0.18	0.39
*Tertiary B/C*	0.08	0.28	0.08	0.27	0.09	0.28
*Tertiary A*	0.77	0.42	0.81	0.40	0.73	0.44
Location						
*Beijing*	0.40	0.49	0.37	0.48	0.43	0.50
*Shanghai*	0.32	0.47	0.36	0.48	0.29	0.45
*Guangzhou*	0.28	0.45	0.26	0.44	0.29	0.45

DP – dual practice, SD – standard deviation, OB/GYN – obstetrics and gynaecology, TCM – Traditional Chinese Medicine, SOE – the state-owned enterprise

*The sample includes doctors working at secondary and tertiary public hospitals in three major Chinese cities in 2020.

†The variable “Online DP” takes the value of one if the doctor provided online medical services on at least one of the four major platforms in December 2020.

‡Other departments include the rest of departments like rehabilitation medicine, stomatology department, and radiology department.

### Prevalence

**Figure 2:**
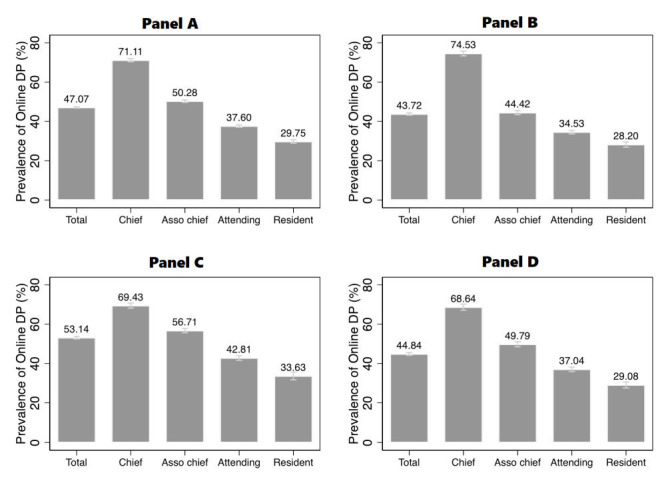
Prevalence of online dual practice in Beijing, Shanghai, and Guangzhou. **Panel A**. The shares of online active public hospital doctors in secondary and tertiary public hospitals in Beijing, Shanghai and Guangzhou. **Panel B**, **C, D.** The shares in three cities, respectively. A doctor is regarded as being engaged in online dual practice when we find the doctor rendering online medical service on any of the four leading online platforms. Despite that the four platforms cover a majority of online doctors, the estimated prevalence is still likely to be the lower bound of the true prevalence. The 95% CIs are calculated based on the Wald method for binomial distributions. DP – dual practice, CIs – confidence intervals

**Figure 2** shows the estimates for the prevalence of online dual practice among public hospital doctors in 2020. At least 47.1% (95% CI = 46.7-47.4%) of public hospital doctors from secondary and tertiary hospitals were engaged in online service provision (**Figure 2**, panel A). The prevalence varied across physician seniority. Chief physicians had the highest prevalence. In these three cities, 71.1% (95% CI = 70.3-71.9%) of chief physicians from secondary and tertiary public hospitals engaged in online service provision on at least one of the four leading platforms, compared with 37.6% (95% CI = 37.0-38.2%) of attending physicians and 29.8% (95% CI = 28.8-30.7%) of resident physicians. Since the data included only four leading telemedicine platforms, these estimates might be underestimates.

The prevalence estimates for online dual practice in Beijing, Shanghai and Guangzhou are presented in **Figure 2**, panels b, c and d. As shown in those panels, the proportion of online dual practitioners was higher in Shanghai than in Beijing and Guangzhou. Those cities had similar patterns of physician seniority distribution, where senior doctors were more likely to be engaged in online dual practice. Specifically, 74.5% (95% CI = 73.3-75.7%), 69.4% (95% CI = 68.2-70.7%) and 68.6% (95% CI = 67.1-70.2%) of chief physicians from public hospitals in Beijing, Shanghai and Guangzhou were engaged in online dual practice. The share of associate chief physicians who engaged in online dual practice in three cities ranged from 44 to 57%. For resident physicians, the prevalence of online dual practice in the three cities ranged from 28 to 34%.

### Factors associated with the engagement of online dual practice

#### Quantitative findings

**Table 3** reports the results of logit regression analysis for engagement in online dual practice. For personal characteristics, online dual practice engagement was significantly associated with gender and education. Male doctors were more likely than their female counterparts to engage in online dual practice, and those with master's or doctoral degrees were more likely to provide online services on private platforms than those with bachelor's degrees. These results remained robust to the inclusion of hospital fixed effects and hospital-department fixed effects. Age, in contrast, did not demonstrate a robust association with participating in online dual practice.

**Table 3:** Odds ratios and marginal effects of factors associated with online dual practice

	Online dual practice*					
	(1)		(2)		(3)	
	OR	AME	OR	AME	OR	AME
**Individual characteristics**						
Male (ref: female)	1.115†	0.023†	1.134†	0.024†	1.132†	0.023†
	(0.020)	(0.004)	(0.022)	(0.004)	(0.023)	(0.006)
Age	0.980§	-0.004§	0.993	-0.001	1.000	-0.000
	(0.011)	(0.002)	(0.014)	(0.003)	(0.018)	(0.003)
Education (ref: college)						
*Master*	1.301†	0.055†	1.195†	0.034†	1.163†	0.028‡
	(0.038)	(0.006)	(0.041)	(0.007)	(0.060)	(0.011)
*Doctoral degree*	2.209†	0.165†	2.144†	0.146†	2.068†	0.136†
	(0.074)	(0.007)	(0.082)	(0.007)	(0.121)	(0.031)
**Professional characteristics**						
Seniority (ref: resident physician)						
*Chief*	9.268†	0.463†	7.283†	0.379†	6.619†	0.353‡
	(2.260)	(0.051)	(2.126)	(0.056)	(2.547)	(0.145)
*Associate chief*	2.539†	0.194†	2.206†	0.151†	2.133†	0.142§
	(0.378)	(0.031)	(0.392)	(0.034)	(0.514)	(0.074)
*Attending*	1.581†	0.095†	1.518†	0.080†	1.487†	0.074‡
	(0.109)	(0.014)	(0.124)	(0.016)	(0.167)	(0.036)
Position (ref: others)						
*Department director*	0.281†	-0.264†	0.262†	-0.256†	0.260†	-0.252†
	(0.012)	(0.008)	(0.011)	(0.008)	(0.021)	(0.053)
*Hospital manager*	0.126†	-0.430†	0.114†	-0.415†	0.112†	-0.409†
	(0.013)	(0.020)	(0.012)	(0.020)	(0.015)	(0.087)
Department (ref: others)						
*Dermatology*	2.209†	0.165†	2.252†	0.155†	n.a.‖	n.a.
	(0.129)	(0.012)	(0.142)	(0.012)		
*Surgery*	2.064†	0.151†	2.197†	0.150†	n.a.	n.a.
	(0.049)	(0.005)	(0.057)	(0.005)		
*Internal medicine*	1.418†	0.073†	1.470†	0.074†	n.a.	n.a.
	(0.035)	(0.005)	(0.039)	(0.005)		
*OB/GYN*	1.348†	0.062†	1.356†	0.058†	n.a.	n.a.
	(0.048)	(0.007)	(0.055)	(0.008)		
*Paediatrics*	1.430†	0.074†	1.516†	0.079†	n.a.	n.a.
	(0.050)	(0.007)	(0.068)	(0.009)		
*TCM*	1.237†	0.044†	1.365†	0.059†	n.a.	n.a.
	(0.033)	(0.006)	(0.052)	(0.007)		
*Mental health*	1.167‡	0.032‡	1.700†	0.101†	n.a.	n.a.
	(0.074)	(0.013)	(0.169)	(0.019)		
**Organisational characteristics**						
Military (ref: government & SOE)	0.099†	-0.480†	n.a.	n.a.	n.a.	n.a.
	(0.005)	(0.010)				
Level of hospital (ref: secondary)						
*Tertiary B & C*	1.191†	0.036†	n.a.	n.a.	n.a.	n.a.
	(0.047)	(0.008)				
*Tertiary A*	1.329†	0.059†	n.a.	n.a.	n.a.	n.a.
	(0.050)	(0.008)				
Location (ref: Beijing)						
*Shanghai*	1.057†	0.011†	n.a.	n.a.	n.a.	n.a.
	(0.021)	(0.004)				
*Guangzhou*	0.883†	-0.026†	n.a.	n.a.	n.a.	n.a.
	(0.018)	(0.004)				
Hospital FEs			Y		Y	
Hospital-department FEs					Y	
Observations	71 944		71 770		70 201	

OR – odds ratio, AME – average marginal effects, ref – reference, OB/GYN – obstetrics and gynaecology, TCM – Traditional Chinese Medicine, SOE – state-owned enterprise, FE – fixed effects, n.a. – no answer, Y – Yes (controlled in the model)

*The variable “online dual practice” takes the value of one if the doctor provided online medical services on at least one of the four major platforms in December 2020. Robust standard errors of estimated coefficients are reported in brackets.

†*P* < 0.01.

‡*P* < 0.05.

§*P* < 0.1.

‖Coefficients for some variables are omitted when controlling for hospital fixed effects and hospital-department fixed effects due to multicollinearity.

Professional characteristics were significantly associated with the engagement of online dual practice among public hospital doctors. Compared to resident physicians, senior doctors were more likely to provide online services on the Internet. The more senior the doctor, the higher the odds of being associated with online dual practice, as demonstrated by the successively decreasing magnitude of coefficients associated with chief doctors, associate chief doctors, and attending doctors. However, management positions were negatively associated with online dual practice. Being a department director or a hospital director was associated with lower odds of online dual practice. These results were robust when we controlled for hospital fixed effects and hospital-department fixed effects. The physician’s clinical department also mattered. Dermatologists and surgeons were more likely to engage in online dual practice, compared to the reference group of physicians from other departments such as rehabilitation medicine, stomatology department and radiology department.

Organisational characteristics were also significantly associated with the engagement of online dual practice. Compared to doctors from hospitals owned by the government and state-owned enterprises (SOEs), doctors at military hospitals were much less likely to be online dual practitioners. Also, public hospital doctors from secondary hospitals were less likely to engage in online dual practice than those from tertiary hospitals. **Table 3** suggests that public hospital doctors in Shanghai were more open to this new form of dual practice than those in Beijing and Guangzhou. These organisational associations remained robust when we separately ran the logit regressions for subsamples from Beijing, Shanghai, and Guangzhou (Table S4 in the **Online Supplementary Document**).

#### Qualitative findings

The qualitative findings triangulated many of the quantitative findings and contributed additional considerations.

##### Personal characteristics

In terms of personal characteristics, some respondents, especially junior doctors, mentioned that financial incentive was an important factor. For example, an attending physician in Guangzhou talked about the high living costs in megacities when explaining her motivations for online dual practice.

*I am in Guangzhou and the living cost here is high. I have a house payment and children to bring up. I can earn some money from the platform.* (P17, female, surgeon, attending physician)

However, more than half of the respondents, most of whom were senior doctors, denied direct remuneration as a significant motivation. They explained that the economic returns from online service provision were limited.

*Online earnings are limited compared to my income from public hospitals. I can earn many times as much as that at hospitals.* (P5, male, surgeon, chief physician)

Some respondents mentioned that they mainly used private platforms to attract complicated cases and optimise patient flows in the physical environment. Such arrangements could increase their earnings while building their national reputation. An interviewee’s statement captured such sentiment well.

*I am in a poor relationship with my department director. Therefore, I have limited opportunities to treat complicated cases and it will influence my career development in the long run. I go to a third-party platform to provide online services and many patients get to know me. These patients can visit me in person when necessary.* (P13, male, internist, attending physician)

The workload at public hospitals was another important factor influencing doctors’ engagement in online dual practice. However, respondents held different opinions regarding its impacts on online engagement. Some doctors mentioned that online platforms can help reduce repetitive and inefficient tasks at public hospitals. Telemedicine appointments can help patients become more prepared for their visits and improve the efficiency of follow-up visits. In contrast, one interviewee said that she was too busy to provide online services in addition to her in-person clinical work.

##### Professional characteristics

For professional characteristics, many respondents confirmed that clinical departments significantly influenced doctors’ inclination to engage in online dual practice. Some of them mentioned that those variations originated from the varying levels of clinical effectiveness via telemedicine. A dermatologist emphasised that she could effectively serve patients on the Internet. In contrast, a dentist, who did not register on private platforms, explained that dentists could only provide limited services via telemedicine because most treatments require in-person services. Moreover, a few doctors argued that variations in online engagement across specialties were attributed to medical resource maldistribution in the physical setting. For example, a paediatrician attributed her engagement in online dual practice to the shortage of paediatricians in China. Her words were as follows:

*Most patients I served on the Internet come from other cities. The number of paediatricians is quite limited in China and most of them locate in large cities. Children and their patients in small cities have a high demand for seeking doctors on the Internet. That is why I would like to engage in online service provision on private platforms.* (P9, female, pediatrician, chief physician)

Two interviewed doctors also mentioned that managerial positions might reduce doctors’ willingness to engage in online dual practice. They further explained that doctors with managerial positions had concerns about conflict of interests, and online dual practice might be controversial. An interviewee gave the following example to illustrate this point:

*I know a doctor who was very active on a third-party platform. He provided a large amount of online medical services and became famous among patients. Online service provision contributed a lot to his career development. However, when he became the director of a top tertiary hospital, he stopped providing online services, for fear of conflicts with his official duties.* (P23, male, internist, chief physician)

##### Organisational characteristics

As for organisational characteristics, many respondents acknowledged that their engagement in online dual practice was largely affected by the underlying attitudes at their affiliated public hospitals. Some respondents mentioned that their affiliated public hospitals, especially military hospitals, explicitly prohibited them from providing online services on private platforms. However, some public hospitals encouraged their doctors to register on private platforms and improve the continuity of hospital care. Those public hospitals believed that online service provision would contribute to improving the hospital's reputation and attract more patients to visit in the physical environment. In the long run, online dual practice became normal in those hospitals and new doctors were encouraged to provide services on private platforms. The following words from two interviewees reflected these opposing attitudes from public hospital managers.

*I have heard that some public hospitals prohibit their doctors from providing online services on third-party platforms. Such restriction partly comes from managers' concern that the hospital may bear some liabilities because of doctors' malpractice on the Internet.* (P27, female, surgeon, associate chief physician)

*Hospital directors encourage us to provide online services on third-party platforms. It can help us better serve patients and improve the continuity of hospital care. Our hospital has served more than 250 000 patients on third-party platforms*. (P31, male, obstetrician and gynecologist, chief physician)

In addition, a few doctors emphasised that quality management on third-party platforms influenced their choice. Before they decided to register on a third-party platform, doctors would assess the quality of online services on this platform. For example, one doctor found that the quality of many services on a platform was problematic, so he decided to leave online service provision. Some doctors, however, were rejected by the platform. Two doctors, for instance, mentioned that their applications were turned down by third-party platforms because of inadequate qualifications.

## DISCUSSION

This study found that online dual practice was prevalent among public hospital doctors from secondary and tertiary hospitals in Beijing, Shanghai, and Guangzhou. Its prevalence among chief physicians in these three cities exceeded 60%, which was much higher than that for junior doctors. The pattern of online dual practice by seniority was similar to what had been previously identified [9]. However, the overall estimates for the magnitude of prevalence in this study were a bit higher than previous estimates for prevalence by doctors from any public hospitals in Beijing, Shanghai, and Guangzhou. There could be two explanations for such a difference. First, this study restricted the sample to public hospital doctors who actively provided in-person outpatient services rather than the whole group of public hospital doctors with medical licenses. We excluded public hospital doctors who engaged in administrative work and hospital management but no longer provided health care services. The other reason was that we only included public hospital doctors from secondary and tertiary hospitals and dropped the sample from primary hospitals due to incomplete information on these doctors. Public hospital doctors from primary hospitals were less likely to engage in online dual practice.

Similar to findings from the literature on dual practice in the physical environment [3,28,29], our quantitative analysis showed that personal, professional, and organisational characteristics were all significantly associated with doctors’ choice to engage in online dual practice. Meanwhile, the subsequent qualitative analysis helped explain and provide depth to these quantitative findings. Four key insights emerged from the qualitative branch. First, financial rewards, both direct earnings from online service provision and indirect economic returns from attracting complicated cases on the Internet, could influence doctors’ inclination to undertake online dual practice. Particularly, attracting online patients to public hospitals became a non-negligible motivation for online dual practice. In addition, doctors’ choices could be affected by their perceived effectiveness of telemedicine and telehealth in improving efficiency and reducing workload. Second, as the quantitative findings showed that the prevalence of online dual practice differed by specialty, qualitative results suggested that the varying prevalence across specialties might be attributed to the effectiveness of telemedicine in substituting in-person care across clinical departments as well as the shortage of medical staff in the in-person setting. Third, public hospital doctors with managerial positions might be concerned about conflicts of interest and potential controversy, according to our interviews. Therefore, some of them chose to terminate online service provision even if they were very active on third-party platforms before they were promoted. Last, hospital ownership and attitudes of managers towards private platforms might also play an important role in influencing doctors’ choices. Therefore, doctors at military hospitals and whose directors took opposing attitudes were much less likely to undertake online dual practice.

Our findings may provide implications for other countries faced with a similar phenomenon of online dual practice, as private telemedicine platforms are quickly growing after the outbreak of the COVID-19 pandemic. First, the financial incentive is an important factor to address doctors’ unwillingness and resistance to uptake telemedicine and telehealth [30,31]. As reported by interviewees, direct and indirect returns from online service provision may influence doctors' engagement in online dual practice. In contrast to the strictly regulated prices for online services within the public hospitals’ telemedicine platforms, the private platforms allow doctors to set their fee schedules flexibly without a volume cap. Besides, doctors like surgeons may indirectly increase their income at public hospitals through attracting complicated cases on the Internet. All these additional earnings thus encourage doctors to increase the supply of medical services after doctors' designated hours and it may contribute to alleviating the health workforce shortage to some extent [1]. This result also partly explains why some Chinese public hospital doctors are unwilling to provide online services at public hospitals’ online platforms.

Second, public hospitals and private online platforms may not always be in a competitive relationship. Online dual practice may improve efficiency via reducing workload, and time constraints may not be a significant factor. Although some individuals felt too busy to take up online dual practice, many interviewees regarded online service provision as a solution to reduce workload and improve efficiency. Furthermore, the more senior doctors who should be busier in fact participate more in online dual practice. It implied that doctors’ perceived effectiveness of telemedicine played an important role in influencing their decisions [32]. Various prevalence estimates by specialties also confirmed this point. These results imply that online service provision by public hospital doctors would sometimes complement to public hospital care. Appropriately integrating online service provision into routine work in the physical environment may be a useful strategy to improve the performance of public hospitals.

Last, the existence of opposing opinions by hospital and department directors regarding whether physicians should pursue online dual practice highlights the importance of clarifying the consequence of online dual practice. A series of studies have investigated the potential impacts of dual practice in the physical health care environment, concluding that dual practice had both positive and negative effects on access, efficiency, and quality [3,10,12,33,34]. However, to the best of our knowledge, few studies except Huang and co-authors, have formally examined the overall impacts of online dual practice and there is no consensus on designing appropriate policy responses to this practice [14]. As there can be mixed impacts according to the theoretical analysis, we recommend that countries that have online dual practice should establish a monitoring and evaluation system for assessing its consequences. This system can be also used to assess how online dual practice affects key indicators of health system performance and to inform dynamic policy adjustments.

Three limitations in this study should be noted for cautionary interpretation and potential future research. First, our empirical analysis was based on public hospital doctors in three Chinese cities. The findings in this study should be treated with caution because factors that influence doctors' online engagement in other regions may be different. Second, we used cross-sectional data in this study, so we were restricted to examining the associative rather than causal relationship. In addition, as online dual practice is an emerging phenomenon, factors that influence doctors’ choices may evolve over time. Third, measuring online dual practice using online data could be imprecise. While the four platforms have large market shares and cover most doctors who provided telemedicine services, the online data set could still be incomplete, resulting in an underestimation of the prevalence of online engagement.

## CONCLUSIONS

Online dual practice was prevalent among doctors at the tertiary and secondary public hospitals in Beijing, Shanghai and Guangzhou. The prevalence of online dual practice varied much across doctors with different personal, professional, and organisational characteristics. Male doctors were more likely than their female counterparts, senior doctors had a higher prevalence than junior ones, and holding managerial positions was negatively associated with online engagement. Clinical specialties, hospital ownership and levels, and geographic locations were also associated factors. The qualitative analysis further suggested that financial returns, perceived effectiveness of telemedicine, and underlying attitudes by hospital directors might be important factors influencing online dual practice engagement. These findings highlight the importance of financial incentives and perceived effectiveness in promoting telemedicine adoption. They also suggest that public hospitals and private online platforms may not always be in a competitive relationship and online dual practice sometimes complements to in-person care provision at public hospitals. As the consequences of online dual practice are complicated, we recommend that relevant countries should establish a monitoring and evaluation system.

## Additional material


Online Supplementary Document
