# Supplementary material for: Factors influencing engagement in online dual practice by public hospital doctors in three large cities: A mixed-methods study in China
Source: J Glob Health. 2023 Sep 22;13:04103. doi: 10.7189/jogh.13.04103 (PMC10514738; doi:10.7189/jogh.13.04103)
Supplement: Online Supplementary Document [file jogh-13-04103-s001.pdf]

ONLINE SUPPLEMENTARY DOCUMENT

Title: Factors influencing engagement in online dual practice by public hospital doctors in three large cities: a mixed-methods study in China

Authors: Duo Xu, Yushu Huang, Sian Tsuei, Hongqiao Fu*, Winnie Yip

* Correspondence to Dr. Hongqiao Fu (hofu90@hsc.pku.edu.cn) Department of Health Policy and Management, School of Public Health, Peking University Health Science Center, Beijing, 100191, China

Contents

Figure S1. Examples of doctors' webpages from Haodf.com Page2

Table S1. Private platforms with public hospital doctors rendering online medical services Page3

Table S2. Leading online healthcare platforms in China Page4

Table S3. Interview guide Page5

Table S4. Odds ratios (OR) of influencing factors by cities Page7

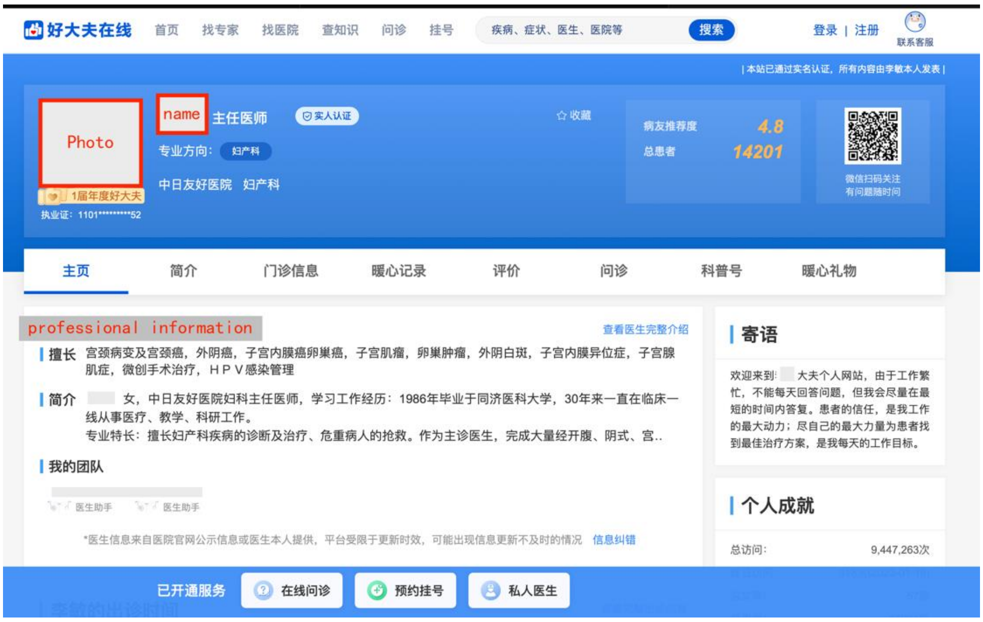

(a) The webpage of a doctor providing online healthcare services

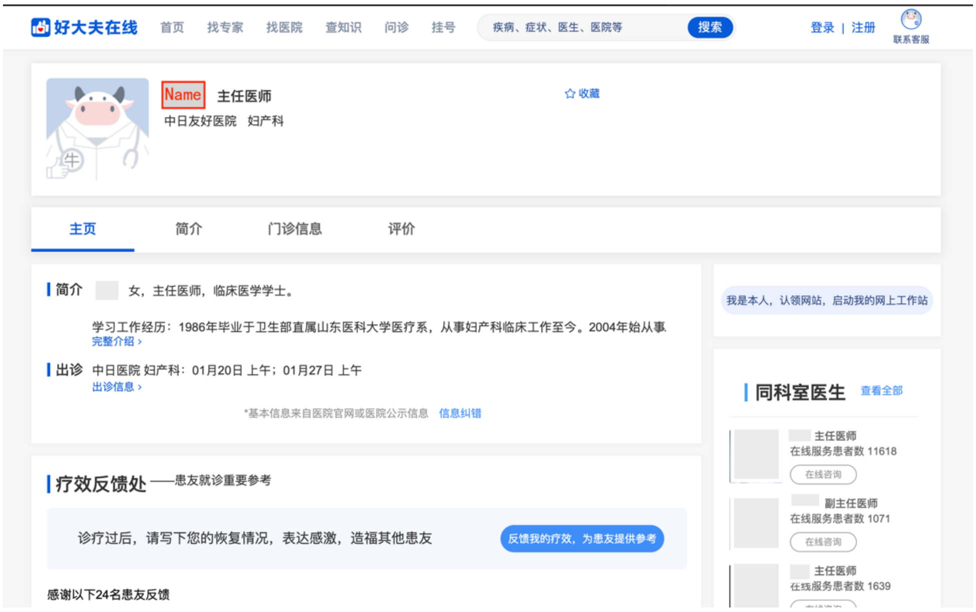

(b) The webpage of a doctor who does not provide online healthcare services

Figure S1: Examples of doctors' webpages from Haodf.com. Haodf.com collects doctors' information from public hospitals and presents it on webpages, regardless of service delivery on Haodf.com platform. The information on these public hospital doctors is updated regularly.

Table S1: Private platforms with public hospital doctors rendering online medical services

Platform	URL	Country	Region
Chunyu Doctor	https://chunyuyisheng.com/	China	East Asia
Haodf.com	https://www.haodf.com/	China	East Asia
Ping'an Health	https://www.pagd.net/	China	East Asia
WeDoctor	https://www.wedoctor.com/	China	East Asia
Doctolib	https://www.doctolib.fr/	France	Western Europe
Credihealth	https://www.credihealth.com/	India	South Asia
DOCGENIE	https://www.docgenie.in/our-doctors/	India	South Asia
Practo	https://www.practo.com/	India	South Asia
TeleMe	https://teleme.co/	Malaysia	Southeast Asia
WhatsDoc Malaysia	https://www.whatsdoc.com/	Malaysia	Southeast Asia
Medifi	https://www.medifi.com/	Philippines	Southeast Asia

Sources: Prepared by authors according to reviews on the official websites of these online healthcare platforms.

Table S2: Leading online healthcare platforms in China

Platform	Main Services	Year of Establishment	Year of Launching Online Healthcare Consultations
Haodf.com	Online consultations, appointment, and disease management	2006	2016
WeDoctor	Online consultations, medicine sales, and medical insurance	2010	2015
Chunyu Doctor	Online consultations and medicine sales	2011	2016
Ping'an Health	Medicine sales, online consultations, and health management	2014	2015
JD Health	Medicine sales, online consultation, and disease management	2019 (Independent from JD.com inc.)	2017 (As a branch of JD.com inc.)
AliHealth	Medicine sales, online consultations	2004 (Acquired by Alibaba in 2014)	2015
Dxy.cn	Online consultation, appointment, medicine sales, and health education	2000	2014
Medlinker	Chronic diseases management	2014	2017
Xiaolu TCM	Traditional Chinese Medicine sales and consultation	2015	2015

Sources: Prepared by authors according to the official websites of these online healthcare platforms.

Table S3: Interview guide

General Information

Participant ID:

Background

Gender/Sex:	Region/Prefecture:
Seniority:	Position:
Level of Hospital:	Specialization:

Introduction

Hello, I'm a researcher from [name of university], and I am conducting a study sponsored by [name of sponsor], on doctors' attitude and opinion on Internet healthcare. I wish to hear from active public hospital doctors providing online services on third-party platforms; may I bother you for an interview?

[Read out the consent form]

All the information you provide will be used for research purpose only, and your name will NOT appear in the reports. Do you mind participating in this interview? Do you have any questions?

General information on online dual practice

1. Do you provide any online healthcare services? If yes, what type(s) of service do you provide on the platforms most often?
2. How many third-party platforms do you provide online services on? Which ones?
3. When did you first start to provide online healthcare services?
4. How many hours do you spend every day on all platforms?
5. Do you know any colleagues who also provide online services? How many?
6. Is there any online platform run by the public hospital you work at?

Overall questions on factors influencing engagement of online dual practice

7. Why do (not) you provide online healthcare services on third-party platforms?
8. Why did you start/stop providing online services?
9. Why did you choose third-party platforms rather than public platforms?

Personal factors influencing engagement of online dual practice

10. Are there any personal factors that impact your decision? (Probe: Income/family...)
    a) How do these factors influence your decision on whether get engaged in online dual practice?

Professional factors influencing engagement of online dual practice

11. Are there any professional factors that impact your decision? (Probe: feature of clinical departments)
    a) How do these factors influence your decision on whether get engaged in online dual practice?

Organizational factors influencing engagement of online dual practice

12. Are there any organizational/institutional factors that impact your decision? (Probe: regulatory constraints)
    a) How do these factors influence your decision on whether get engaged in online dual practice?
13. Does your institution impose regulations on online healthcare services?
    a) Are the regulations imposed by your department director?

b) Are the regulations imposed by your hospital managers?<br>c) What do you think of the regulations?
**Perception on online dual practice**<br>14. Have you heard of government regulations on online healthcare services?<br>a) What do you think of the regulations?<br>15. Do you have any other concerns or comments on public doctors providing healthcare services online?
**Additional questions**<br>Thank you very much for your time. Is there any other information you would like to share, or any recommendations you would like to make to the policymakers?
**General Comments**

Table S4: Odds ratios (OR) of influencing factors by cities

Dep. Var	Online dual practice†		
Location	Beijing	Shanghai	Guangzhou
	(1)	(2)	(3)
**Individual Characteristics**			
Male (*ref: Female*)	1.119***	1.088***	1.149***
	(0.036)	(0.034)	(0.039)
Age	0.985	0.939***	0.913***
	(0.025)	(0.020)	(0.018)
Education (*ref: College*)			
Master	1.392***	0.979	1.854***
	(0.070)	(0.049)	(0.109)
Doctoral Degree	2.442***	1.797***	2.873***
	(0.145)	(0.103)	(0.186)
**Professional Characteristics**			
Seniority (*ref: Resident Physician*)			
Chief	68.563***	12.932***	31.349***
	(37.843)	(5.903)	(13.457)
Associate Chief	2.118**	4.858***	6.504***
	(0.675)	(1.392)	(1.722)
Attending	1.472***	2.196***	2.392***
	(0.218)	(0.293)	(0.287)
Position (*ref: Non*)			
Department Director	0.023***	0.841**	0.412***
	(0.003)	(0.059)	(0.033)
Hospital Director	0.009***	0.404***	0.161***
	(0.002)	(0.069)	(0.030)
Department (*ref: Others‡*)			
Dermatology	2.414***	1.726***	2.890***
	(0.238)	(0.163)	(0.344)
Surgery	2.181***	1.978***	2.057***
	(0.086)	(0.080)	(0.095)
Internal Medicine	1.413***	1.359***	1.524***
	(0.055)	(0.057)	(0.073)
OB/GYN	1.247***	1.890***	0.989
	(0.079)	(0.117)	(0.063)
Pediatrics	1.571***	1.806***	1.065
	(0.099)	(0.116)	(0.064)
TCM	1.401***	1.304***	1.007
	(0.065)	(0.064)	(0.048)
Mental Health	1.044	1.030	1.684***
	(0.111)	(0.112)	(0.200)
**Organizational Characteristics**			
Military (*ref: Government & SOE*)	0.101***	0.030***	0.523***
	(0.006)	(0.005)	(0.059)
Level of Hospital (*ref: Secondary*)			
Tertiary B & C	1.224***	1.378***	1.545***
	(0.075)	(0.105)	(0.055)
Tertiary A	1.479***	1.404***	1.490***
	(0.077)	(0.085)	(0.081)
Observations	28,834	23,192	19,918

OB/GYN – obstetrics and gynecology, TCM – Traditional Chinese Medicine, SOE – the state-owned enterprise.

†The sample and the method of estimation is the same as those in column 1 of Table 3. Robust standard errors of coefficients are reported in brackets. *** p<0.01, ** p<0.05, * p<0.1.

‡Others include departments like rehabilitation medicine, stomatology department, and radiology department.
